# P-1981. MMCD score for predicting kidney replacement therapy in hospitalized patients with COVID-19 from 2020 to 2022

**DOI:** 10.1093/ofid/ofae631.2139

**Published:** 2025-01-29

**Authors:** Vanessa das Graças José Ventura, Polianna Delfino-Pereira, Magda Carvalho Pires, Alexandre Vargas Schwarzbold, Alzira de Oliveira Jorge, Angélica Gomides dos Reis Gomes, Bruno Porto Pessoa, Christiane Correa Rodrigues Cimini, Daniela Ponce, Danyelle Romana Alves Rios, Evelim Thainara Conceição de Oliveira, Fernando Anschau, Fernando Graça Aranha, Flávio de Azevedo Figueiredo, Frederico Bartolazzi, Genna Maira Santos Grizende, Joanna D’arc Lyra Batista, Karen Brasil Ruschel, Luanna Silva Monteiro Menezes, Lucas Rocha Valle, Luiza Marinho Motta Santa Rosa, Marcelo Carneiro, Pedro Gibson Paraíso, Saionara Cristina Francisco, Silvana Mangeon Mereilles Guimarães, Tatiani Oliveira Fereguetti, Katia de Paula Farah, Milena Soriano Marcolino

**Affiliations:** Universidade Federal de Minas Gerais, Belo Horizonte, Minas Gerais, Brazil; UFMG, Belo Horizonte, Minas Gerais, Brazil; Federal University of Minas Gerais, Belo Horizonte, Minas Gerais, Brazil; Hospital Universitário de Santa Maria, Santa Maria, Rio Grande do Sul, Brazil; UFMG, Belo Horizonte, Minas Gerais, Brazil; Hospitais da Rede Mater Dei., Belo Horizonte, Minas Gerais, Brazil; Hospital Júlia Kubitschek, Belo Horizonte, Minas Gerais, Brazil; Universidade Federal dos Vales do Jequitinhonha e Mucuri (UFVJM), Teólifo Otoni, Minas Gerais, Brazil; Botucatu Medical School, Universidade Estadual Paulista "Júlio de Mesquita Filho", Botucatu, Sao Paulo, Brazil; Hospital São João de Deus, Divinopolis, Minas Gerais, Brazil; Hospital Universitário Canoas, Canoas, Rio Grande do Sul, Brazil; Hospital Nossa Senhora da Conceição, Porto Alegre, Rio Grande do Sul, Brazil; SOS Cardio, Florianópolis, Santa Catarina, Brazil; UFMG, Belo Horizonte, Minas Gerais, Brazil; Hospital Santo Antônio, Curvelo, Minas Gerais, Brazil; Santa Casa de Belo Horizonte, Belo Horizonte, Minas Gerais, Brazil; Hospital Regional do Oeste, Chapecó, Santa Catarina, Brazil; Hospital Universitário de Canoas, Canoas, Rio Grande do Sul, Brazil; Federal University of Minas Gerais (UFMG), Belo Horizonte, Minas Gerais, Brazil; Universidade Federal de Minas Gerais, Belo Horizonte, Minas Gerais, Brazil; FACULDADE DE CIÊNCIAS MÉDICAS DE MINAS GERAIS, Belo Horizonte, Minas Gerais, Brazil; HOSPITAL SANTA CRUZ, Santa Cruz do Sul, Minas Gerais, Brazil; Instituto Orizonti, Belo Horizonte, Minas Gerais, Brazil; HOSPITAL METROPOLITANO DR. CÉLIO DE CASTRO, Belo Horizonte, Minas Gerais, Brazil; HOSPITAL SEMPER, Belo Horizonte, Minas Gerais, Brazil; Hospital Eduardo de Menezes, Belo Horizonte, Minas Gerais, Brazil; UFMG, Belo Horizonte, Minas Gerais, Brazil; UFMG, Belo Horizonte, Minas Gerais, Brazil

## Abstract

**Background:**

Acute kidney injury (AKI) requiring kidney replacement therapy (KRT) in its most severe forms is a significant complication of patients with COVID-19. The development of a risk score to predict KRT requirement could optimize health resource allocation. Thus, this study aimed to develop and validate a score to predict the KRT requirement in hospitalized patients with COVID-19 from 2020 to 2022.

Area under the receiver operating characteristic curve for the 2021-2022 validation of MMCD score

**Figure:**
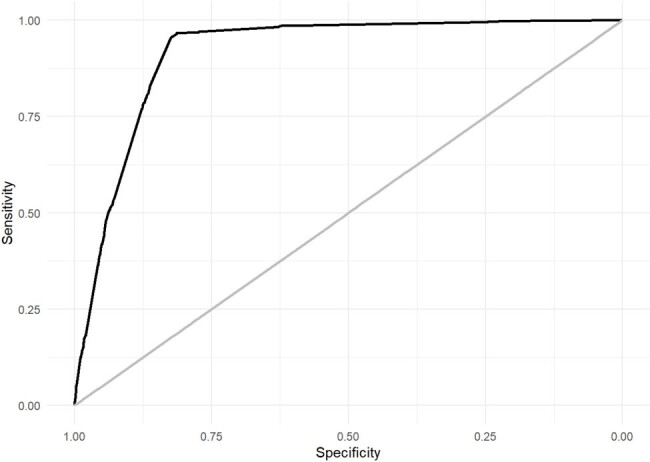

**Methods:**

This is a multicenter retrospective cohort of consecutive patients hospitalized for COVID-19, confirmed by laboratory tests, in 40 Brazilian hospitals, from March 2020 to July 2022. Patients under 18 years old, pregnant, in palliative care, or in dialysis therapy at admission were excluded from the study. Predictor variables were selected using generalized additive models (GAM) and the least absolute shrinkage and selection operator (LASSO) regression was used for score derivation. The score was developed in the period from March to July 2020, with temporal and geographic validation from July to September 2020 and new temporal validation from March 2021 to July 2022. The performance of the score was evaluated by the area under the receiver operating characteristic curve (AUROC, with a 95% confidence interval), graphical analysis with intercept and slope test, and Brier score.

Calibration for the validation of MMCD score

**Figure:**
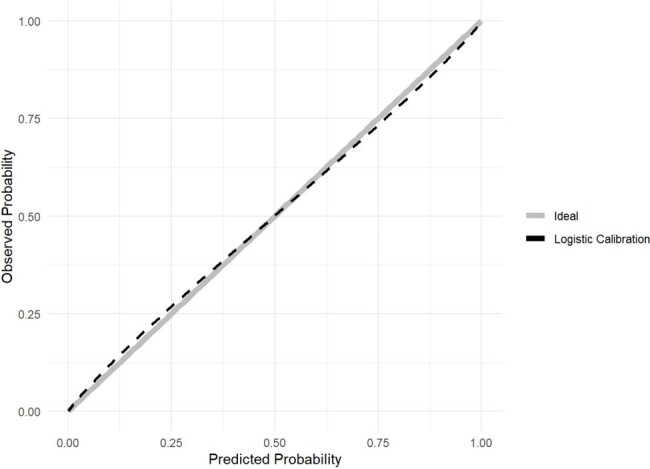

**Results:**

The study included 3,680 patients in the development sample, 1,532 in the 2020 temporal validation, 1,378 in the geographic validation, and 9,473 in the 2021-2022 temporal validation. Four predictors were identified: mechanical ventilation at any time during hospitalization, male sex, admission creatinine, and diabetes mellitus. The score named MMCD showed excellent performance in the derivation and validations cohorts (development AUROC 0.929, CI 0.918–0.939, Brier 0.057; 2020 temporal validation AUROC 0.927, CI 0.911–0.941, Brier 0.056; 2020 geographic validation AUROC 0.819, CI 0.792–0.845, Brier 0.122; 2021/2022 temporal validation AUROC 0.916, CI 0.909-0.924, Brier 0.057).

**Conclusion:**

The MMCD showed excellent predictive ability for KRT in different phases of the pandemic, which can contribute to support more assertive decisions in the allocation of care resources, in addition to efficient clinical management.

**Disclosures:**

All Authors: No reported disclosures

